# Complete genomes of *Mucilaginibacter sabulilitoris* SNA2 and *Mucilaginibacter* sp. cycad4: microbes with the potential for plant growth promotion

**DOI:** 10.1128/mra.00626-24

**Published:** 2024-11-27

**Authors:** Ann M. Hirsch, Ethan Humm, Liudmilla Rubbi, Giorgia Del Vecchio, Sung Min Ha, Matteo Pellegrini, Robert P. Gunsalus

**Affiliations:** 1 Department of Molecular, Cell, and Developmental Biology, University of California, Los Angeles, California, USA; 2 Department of Microbiology, Immunology, and Molecular Genetics, University of California, Los Angeles, California, USA; 3 Department of Integrative Biology and Physiology, University of California, Los Angeles, California, USA; 4 UCLA DOE Institute, University of California, Los Angeles, California, USA; University of Strathclyde, Glasgow, United Kingdom

**Keywords:** plant growth promotion, endophytes, *Mucilaginibacter*, genomes

## Abstract

*Mucilaginibacter* species have been isolated from various environments, often in association with plants. Here, we report the complete genomes of *Mucilaginibacter sabulilitoris* SNA2 and *Mucilaginibacter* sp. cycad4. The former is the first available for that species, and based on 16S sequence analysis, the latter strain is likely a new species.

## ANNOUNCEMENT

*Mucilaginibacter* species have been isolated from sources including soil (1, 2), marine sand (3), water (4, 5), and plant tissue (6). Members of the genus have been reported to promote plant growth (1) and degrade pollutants (2). *Mucilaginibacter* sp. cycad4 and *Mucilaginibacter sabulilitoris* SNA2 were obtained from a surface-sterilized *Encephalartos arenarius* (Alexandria cycad) coralloid root and a *Medicago polymorpha* (burr medic) root nodule, respectively, in separate studies of endophytic microbes in plants growing in the Mildred E. Mathias Botanical Garden, UCLA. Surface-sterilized plant tissues were aseptically crushed with a mortar and pestle according to Youseif et al. (7), and serial dilutions were spread on agar plates and incubated at 30°C for 1 week. *M*. sp. cycad4 was picked from a Bristol’s agar plate (8), whereas *M. sabulilitoris* SNA2 was picked from a nutrient agar plate (BD 213000). Colonies were streak plated to obtain pure cultures and glycerol stocks prepared for storage at −80°C . Initial identifications were by 16S rRNA gene PCR and sequencing as described by Khan et al. (9) with subsequent whole-genome analysis.

The *Mucilaginibacter* strains were obtained from the Hirsch culture collection and cultivated in TY medium aerobically at 30°C. DNA was extracted using the Quick-DNA HMW Magbead Kit (Zymo Research) per the manufacturer’s instructions. DNA was fragmented using Covaris gTubes following the manufacturer’s instructions (four passes at 7,000 rpm through the gTube orifice), and the average size of the sheared gDNA was checked at the TapeStation 4200 (Agilent). Multiplexed microbial libraries were prepared using the PacBio SMRTbell prep kit 3.0 together with the SMRTbell barcoded adapters 3.0 according to the PacBio protocol. Final whole-genome libraries were not size-selected but simply purified via a standard procedure using 1× SMRTbell cleanup beads. DNA sequencing was performed using the PacBio Sequel IIe platform. Demultiplexing and adapter trimming were done using Lima v2.9.0 (https://github.com/pacificbiosciences/barcoding). High-quality reads were assembled by Canu v2.2 (10). Assembled genomes were further refined by Circlator v1.5.5 (11) to identify circular contigs, remove redundant non-circular contigs, and rotate circular contigs to start with *dnaA*, resulting in a circular chromosome with no plasmids for each strain. A completeness check was performed by CheckM v1.0.18 (12), and the N50 quality was determined by Assembly stats ver1.01 (https://github.com/sanger-pathogens/assembly-stats). Genome open reading frame calling and annotation were performed by NCBI’s PGAP v6.6 (13).

Properties of each genome are shown in Table 1. Genomic 16S gene sequences of *M. sabulilitoris* SNA2 and *M*. sp. cycad4 strains were compared to the published 16S sequence of *M. sabulilitoris* SMS-12^T^ and *Mucilaginibacter celer* HYN0043^T^, respectively, indicating that the latter is a new species (14, 15). Average nucleotide identity analysis performed using the Ezbiocloud ANI Calculator (16) confirmed this conclusion.

**TABLE 1:** *M. sabulilitoris* SNA2 and *M.* sp. cycad4 genome information

	***M. sabulilitoris*** SNA2	*M.* sp. cycad4
Topology	Circular	Circular
Size (bp)	7,552,305	7,111,297
GC%	42%	43%
Coverage	32.0×	34.0×
Total raw reads	255,280	201,498
Average read length	6,553.07 bp	4,653.93 bp
Raw reads N50	7,077 bp	5,420 bp
High-quality reads	23,238	30,176
Completeness	96.67%	97.62%
Contigs N50	7,552,305 bp	7,111,297
Protein-coding genes	6,289	5,817
16S number	2	3
tRNA number	48	53
Highest 16S identity	99.03%^*a*^	98.26%^*b*^
Highest ANI	78.61%^*c*^	88.29%^*d*^
Isolation date	25 April 2018	20 December 2019
Collection site coordinates	34.066250, –118.441083	34.066004, –118.441566

^*a*^ Genomic 16S to *M. sabulilitoris* SMS-12^T^ (NR_118395.1).

^*b*^ Genomic 16S to *M. celer* HYN0043^T^ (NR_174213.1).

^*c*^ Whole genome to *M. lappiensis* ATCC BAA-1855^T^ (GCA_900155965.1; whole genome of *M. sabulilitoris* SMS-12^T^ unavailable).

^*d*^ Whole genome to *M. rubeus* CGMCC 1.15913^T^ (GCA_014643835.1).

The finished genome sequences each possess potential plant growth-promoting traits based on DNA sequence analysis. Both encode the enzyme 1-aminocyclopropane-1-carboxylic acid (ACC) deaminase (acdS), which may improve plant growth by reducing levels of stress ethylene, and genes for acetoin and trehalose biosynthesis. Additionally, various hydrolytic enzymes are encoded, including xylan 1,4-beta-xylosidase, endoglucanase, polygalacturonase, and alpha-amylase.

## Data Availability

The raw sequencing reads have been deposited under the SRA accession numbers SRR26961106 and SRR26961105 for SNA2 and cycad4, respectively. The assembled genomes are listed under the GenBank accession numbers CP139558 and CP139559 for SNA2 and cycad4, respectively.

## References

[1] Madhaiyan M, Poonguzhali S, Lee J-S, Senthilkumar M, Lee KC, Sundaram S. 2010. Mucilaginibacter gossypii sp. nov. and Mucilaginibacter gossypiicola sp. nov., plant-growth-promoting bacteria isolated from cotton rhizosphere soils. Int J Syst Evol Microbiol 60:2451–2457. doi:10.1099/ijs.0.018713-0 19946048

[2] You XY, Liu JH, Tian H, Ding Y, Bu QY, Zhang KX, Ren GY, Duan X. 2022. Mucilaginibacter phenanthrenivorans sp. nov., a novel phenanthrene degradation bacterium isolated from Wetland Soil. Curr Microbiol 79:382. doi:10.1007/s00284-022-03085-z 36329315

[3] Kang CH, Jung YT, Yoon JH. 2013. Mucilaginibacter sabulilitoris sp. nov., isolated from marine sand in a firth. Int J Syst Evol Microbiol 63:2865–2871. doi:10.1099/ijs.0.045989-0 23315407

[4] Sheu SY, Xie YR, Chen WM. 2019. Mucilaginibacter limnophilus sp. nov., isolated from a lake. J Microbiol 57:967–975. doi:10.1007/s12275-019-9146-z 31463791

[5] Chen WM, Hsieh TY, Sheu SY. 2018. Mucilaginibacter amnicola sp. nov., isolated from a freshwater creek. Int J Syst Evol Microbiol 68:394–401. doi:10.1099/ijsem.0.002518 29219806

[6] Akter S, Huq MA. 2020. Mucilaginibacter corticis sp. nov., isolated from bark of Pinus koraiensis. Antonie Van Leeuwenhoek 113:491–498. doi:10.1007/s10482-019-01358-5 31741188

[7] Youseif SH, El-Megeed FHA, Salous MS, Mohamed AH. 2023. Streptomyces biostimulants: an effective sustainable approach to reduce inorganic N input and maintain high yield of wheat crop in different soil types. J Appl Microbiol 134:lxad156. doi:10.1093/jambio/lxad156 37468449

[8] Bold HC. 1949. The morphology of Chlamydomonas chlamydogama, sp. nov. Bull Torrey Bot Club 76:101. doi:10.2307/2482218

[9] Khan N, Martínez-Hidalgo P, Humm EA, Maymon M, Kaplan D, Hirsch AM. 2020. Inoculation with a microbe isolated from the Negev Desert enhances corn growth. Front Microbiol 11:1149. doi:10.3389/fmicb.2020.01149 32636811 PMC7316896

[10] Koren S, Walenz BP, Berlin K, Miller JR, Bergman NH, Phillippy AM. 2017. Canu: scalable and accurate long-read assembly via adaptive k -mer weighting and repeat separation . Genome Res 27:722–736. doi:10.1101/gr.215087.116 28298431 PMC5411767

[11] Hunt M, Silva ND, Otto TD, Parkhill J, Keane JA, Harris SR. 2015. Circlator: automated circularization of genome assemblies using long sequencing reads. Genome Biol 16:294. doi:10.1186/s13059-015-0849-0 26714481 PMC4699355

[12] Parks DH, Imelfort M, Skennerton CT, Hugenholtz P, Tyson GW. 2015. CheckM: assessing the quality of microbial genomes recovered from isolates, single cells, and metagenomes. Genome Res 25:1043–1055. doi:10.1101/gr.186072.114 25977477 PMC4484387

[13] Tatusova T, DiCuccio M, Badretdin A, Chetvernin V, Nawrocki EP, Zaslavsky L, Lomsadze A, Pruitt KD, Borodovsky M, Ostell J. 2016. NCBI prokaryotic genome annotation pipeline. Nucleic Acids Res 44:6614–6624. doi:10.1093/nar/gkw569 27342282 PMC5001611

[14] Lee I, Chalita M, Ha SM, Na SI, Yoon SH, Chun J. 2017. ContEst16S: an algorithm that identifies contaminated prokaryotic genomes using 16S RNA gene sequences. Int J Syst Evol Microbiol 67:2053–2057. doi:10.1099/ijsem.0.001872 28639931

[15] Yoon SH, Ha SM, Kwon S, Lim J, Kim Y, Seo H, Chun J. 2017. Introducing EzBioCloud: a taxonomically united database of 16S rRNA gene sequences and whole-genome assemblies. Int J Syst Evol Microbiol 67:1613–1617. doi:10.1099/ijsem.0.001755 28005526 PMC5563544

[16] Yoon SH, Ha SM, Lim J, Kwon S, Chun J. 2017. A large-scale evaluation of algorithms to calculate average nucleotide identity. Antonie Van Leeuwenhoek 110:1281–1286. doi:10.1007/s10482-017-0844-4 28204908

